# Supplementary material for: Revealing the Sequence Characteristics and Molecular Mechanisms of ACE Inhibitory Peptides by Comprehensive Characterization of 160,000 Tetrapeptides
Source: Foods. 2023 Apr 7;12(8):1573. doi: 10.3390/foods12081573 (PMC10137938; doi:10.3390/foods12081573)
Supplement: Supplementary file 1 [file foods-12-01573-s001.zip › foods-2308038-supplementary.pdf]

Supporting Information

Revealing the Sequence Characteristics and Molecular Mechanisms of ACE Inhibitory Peptides by Comprehensive Characterization of 160,000 Tetrapeptides

Mingzhe Ma [1], Yinghui Feng [1], Yulu Miao [1], Qiang Shen [1], Shuting Tang [2], Juan Dong [2], John Z. H. Zhang [1,3,4,*] and Lujia Zhang [1,3,*]

1 Shanghai Engineering Research Center of Molecular Therapeutics & New Drug Development, School of Chemistry and Molecular Engineering, East China Normal University, Shanghai 200062, China; 52204300067@stu.ecnu.edu.cn (M.M.); yhfeng@chem.ecnu.edu.cn (Y.F.); ylm695@163.com (Y.M.); 13661657406@163.com (Q.S.)

2 School of Food Science and Technology, Shihezi University, Shihezi 832000, China; 15729932216@163.com (S.T.); dongjuanvv@126.com (J.D.)

3 NYU-ECNU Center for Computational Chemistry at NYU Shanghai, Shanghai 200062, China

4 Department of Chemistry, New York University, New York, NY 10003, USA

* Correspondence: john.zhang@nyu.edu (J.Z.H.Z.); ljzhang@chem.ecnu.edu.cn (L.Z.)

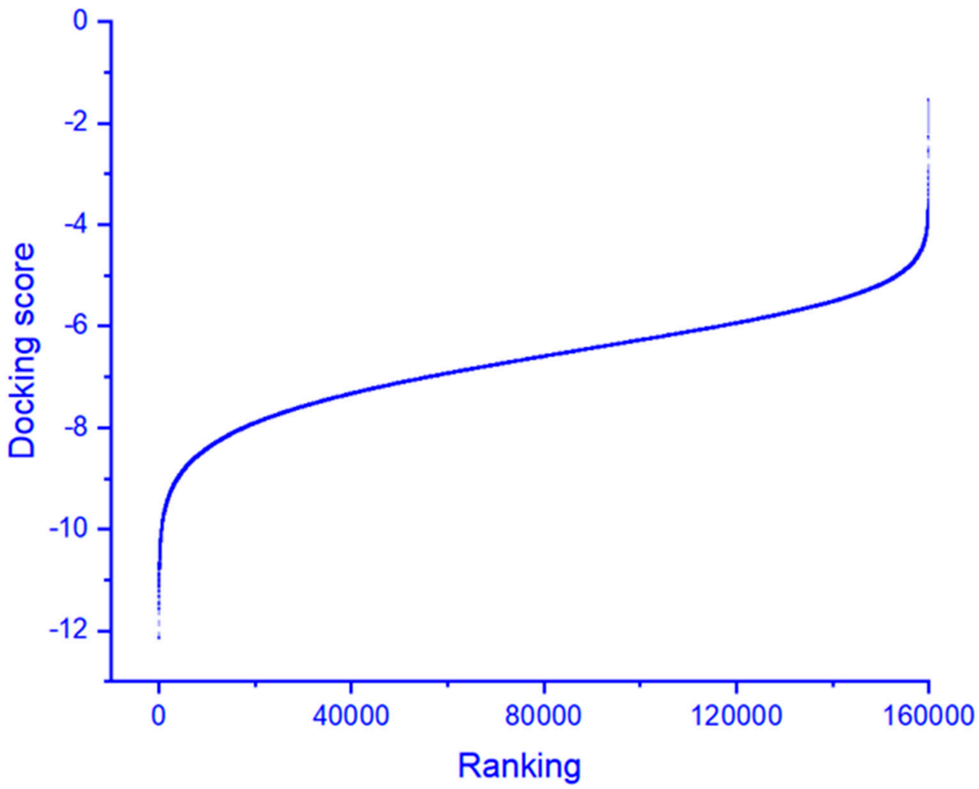

Figure S1. The graph of tetrapeptide docking scoring results and ranking.

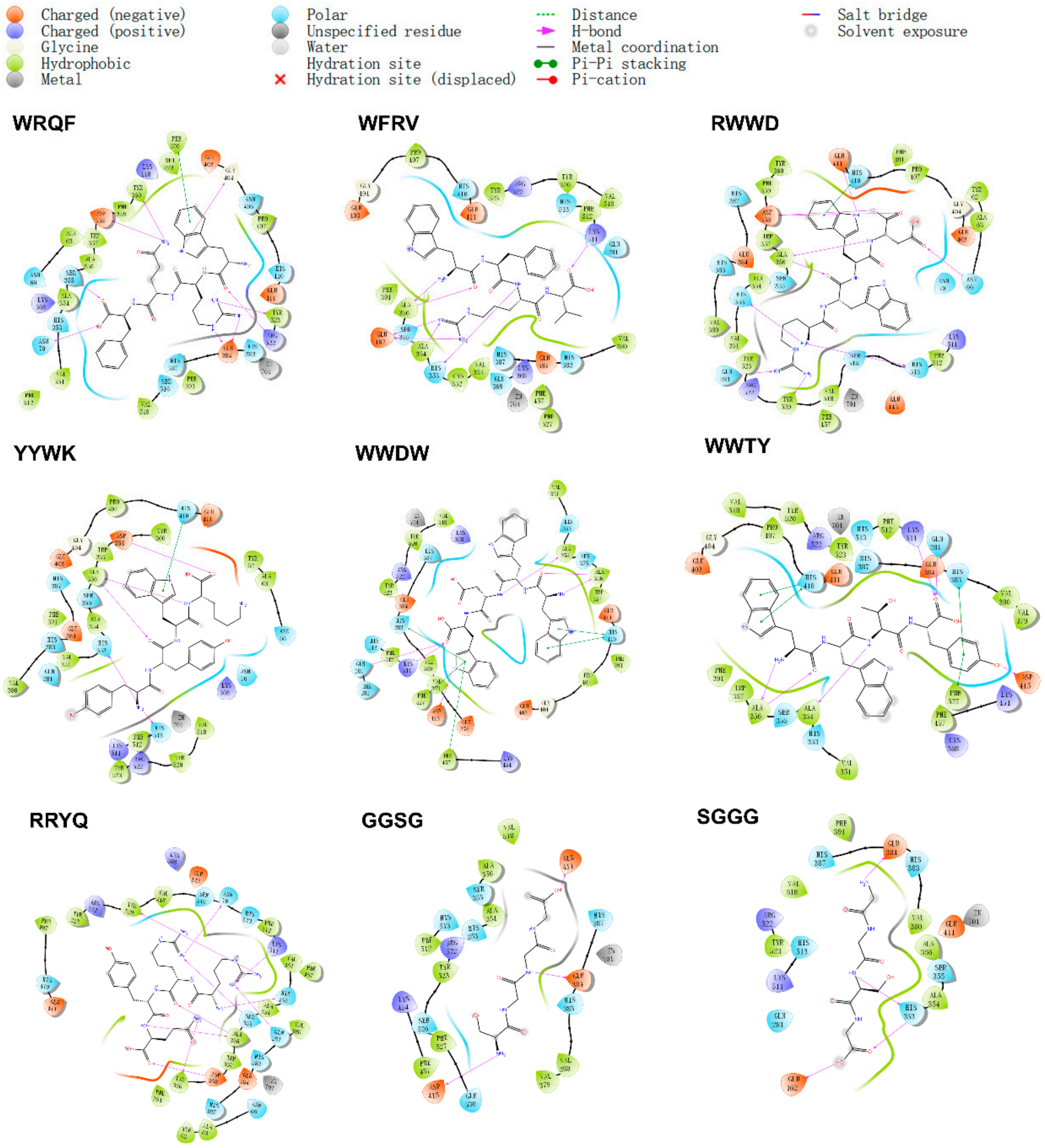

Figure S2. The 2D interaction map of ACE with top 4-10 and last 2 tetrapeptides.

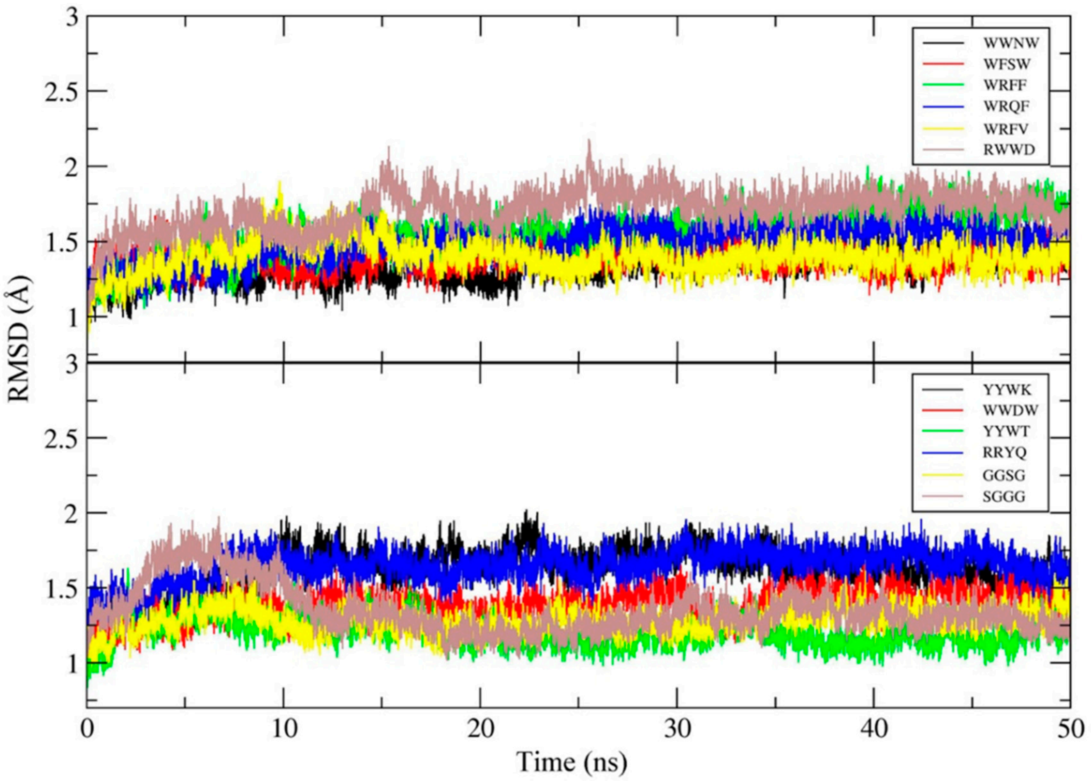

Figure S3. The RMSD plots of the top 10 and the last 2 ACE-tetrapeptide complexes.

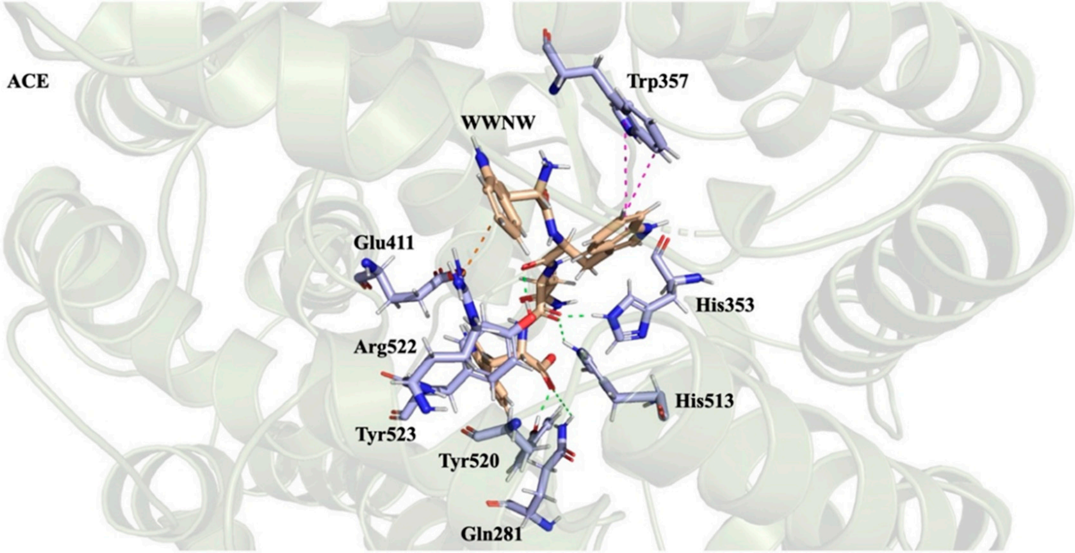

Figure S4. The interaction map of 8 hotspot residues of ACE with WWNW.

```
SK-WT.txt      IKIEFSKEQQDEFKEAFLLYDRTGDSKITLSQVGDVLRALGTNPTNAEVKK     51
Mutant1.txt    IKIEWSKEQQDEFKEAFLLYDRTGDSKITLSQVGDVLRALGTWPTNAEVKK     51
Mutant2.txt    IKIEWSKEQQDEFKEAFLLYDRWGDSKITLSQVGDVLRALGTNWTNAEVKK     51

SK-WT.txt      VLGNPSNEEMNAKKIEFEQFLPMLQAISNNKDQGTYEDFVEGLRVFDKEGN    102
Mutant1.txt    VLGNPSWEEMNAKKIEFEQFLPMLQAIWNNKDQGTYEDFVEGLRVFDKEGN    102
Mutant2.txt    VLGNPWNEEMNAKKIEFEQWLPMLQAISNNKDQGTYEDFVEGLRVWDKEGN    102

SK-WT.txt      GTVMGAELRHVLATLGEKMKEEEVEALMAGQEDSNGCINYEAFVKHIMS      151
Mutant1.txt    GTVMGAELRHVLATLWEKMKEEEVEALMAGQEDSNGCIWYEAFVKHIMS      151
Mutant2.txt    GTVMGAELRHVLATLGEKMKEEEVEALMAGQEDWNGCINYEAFVKHIMW      151
```

Figure S5. Sequence comparison of rabbit-derived skeletal myosin chain C (wild type) and tryptophan-containing mutants.

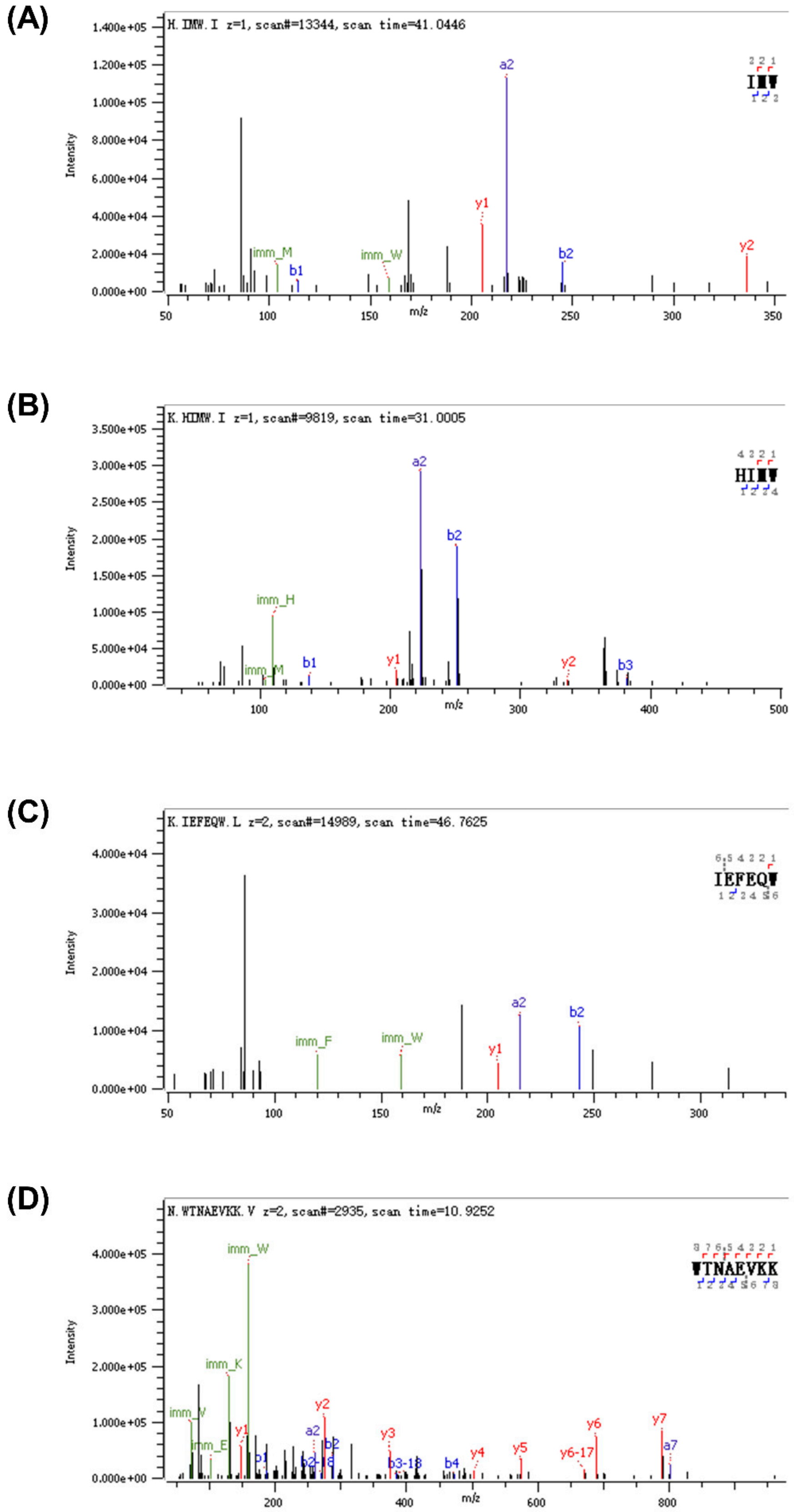

Figure S6. Secondary mass spectra of tripeptide (A) IMW; (B) HIMW; (C) IEFEQW; (D) WTNAEVKK.

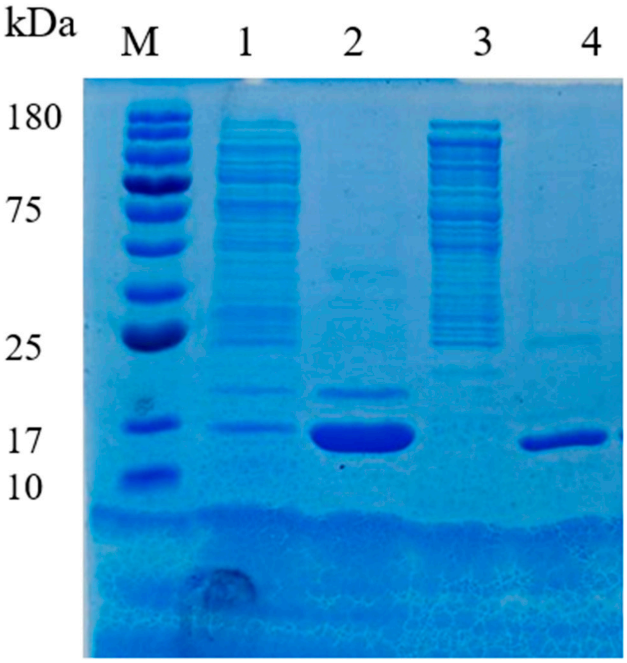

Figure S7. SDS-PAGE of purified proteins of mutants. 1-2: mut 1; 3-4: mut 2.

Table S1. The hotspot residues of ACE-tetrapeptide complexes with scores rank 1-5 calculated by AS-GBIE method

Mutation	ACE-WWNW	ACE-WFSW	ACE-WRFF	ACE-WRQF	ACE-WRFV
			$\Delta\Delta G_{bind}$(kcal/mol)		
Q281A	-1.50±0.19	-1.35±0.13	/	/	-2.83±0.11
H353A	-2.79±0.50	-2.44±0.11	-2.22±1.00	/	-1.87±1.10
S355A	/	-1.02±0.15	/	/	/
W357A	-1.50±0.22	/	-1.21±0.27	-2.28±2.67	/
Y360A	/	/	/	-1.19±0.97	/
E384A	-1.81±1.05	/	/	/	-1.18±0.58
H387A	/	/	/	-1.20±0.73	/
F391A	/	/	/	-1.00±0.19	/
E403A	/	/	/	-1.58±0.77	/
H410A	/	/	/	-1.78±1.06	/
E411A	-1.28±0.19	-1.04±0.04	/	/	-1.63±0.19
F512A	-1.33±0.14	-1.12±0.08	-1.29±0.67	/	/
H513A	-2.22±0.07	-2.40±0.23	-1.12±1.06	/	/
V518A	-1.38±0.13	/	/	/	/
Y520A	-1.61±0.16	-1.39±0.07	/	/	-2.58±01.2
R522A	-2.26±0.46	-1.88±0.84	/	/	-1.74±1.31
Y523A	-3.96±0.50	-4.15±0.06	/	/	-1.85±0.48

Table S2. The hotspot residues of ACE-tetrapeptide complexes with scores rank 6-10 calculated by AS-GBIE method

Mutation	ACE-RWWD	ACE-YYWK	ACE-WWDW	ACE-WWTY	ACE-RRYQ
			$\Delta\Delta G_{bind}$(kcal/mol)		
Q281A	/	/	-1.14±0.09	-1.92±0.79	/
H353A	-2.20±1.09	-2.10±0.35	-2.65±0.21	-2.25±0.35	/
W357A	/	-2.68±1.35	-2.44±0.36	-1.93±0.69	-1.87±1.32
Y360A	/	-1.43±0.45	/	/	/
H383A	/	/	/	/	-2.52±0.28
E384A	/	/	-1.38±1.68	-1.61±0.30	/
F391A	/	-1.52±0.69	/	/	/
H410A	/	-1.53±0.68	-1.29±0.08	-.131±0.32	/
E411A	/	/	/	-1.07±0.21	-2.42±0.60
F512A	/	/	/	-1.08±0.18	/
H513A	-1.18±0.37	-1.47±0.39	-2.53±0.28	-2.43±0.14	/
V518A	/	/	-1.37±0.31	-1.60±0.33	/
Y520A	/	/	-1.77±0.21	-1.51±0.37	/
R522A	/	-1.01±0.12	-2.60±0.31	-1.59±0.17	-1.06±1.62
Y523A	-1.22±0.75	/	-3.06±0.29	-4.47±0.30	-1.66±1.03

Table S3 Binding free energy of ACE-tetrapeptide complexes calculated by AS-GBIE method

WWNW	Md1			Md2			Md3		
Mutation	ΔH	$-T\Delta S$	ΔG_{bind}	ΔH	$-T\Delta S$	ΔG_{bind}	ΔH	$-T\Delta S$	ΔG_{bind}
N66A	-0.09	0.06	-0.03	-0.25	0.06	-0.19	-0.14	0.06	-0.08
N70A	-0.10	0.49	0.39	-0.68	0.49	-0.19	-0.25	0.10	-0.15
E143A	-0.32	0.04	-0.28	-0.41	0.04	-0.37	-0.41	0.04	-0.37
Q281A	-2.05	0.29	-1.77	-1.60	0.29	-1.31	-2.12	0.68	-1.44
T282A	-0.55	0.09	-0.46	-0.53	0.09	-0.44	-0.58	0.10	-0.48
V351A	-0.07	0.02	-0.05	-0.22	0.02	-0.19	-0.09	0.00	-0.08
H353A	-4.40	1.59	-2.81	-3.75	1.59	-2.16	-4.47	1.08	-3.39
S355A	-0.07	-0.13	-0.20	-0.40	-0.13	-0.53	-0.38	-0.06	-0.43
W357A	-2.45	0.64	-1.81	-2.04	0.64	-1.40	-1.91	0.61	-1.30
D358A	-0.26	0.02	-0.24	-0.25	0.02	-0.24	-0.27	0.01	-0.26
Y360A	-0.04	0.05	0.01	-0.12	0.05	-0.07	-0.07	0.02	-0.05
K368A	0.03	0.01	0.04	-0.04	0.01	-0.02	0.02	0.01	0.03
E376A	0.03	0.01	0.04	0.03	0.01	0.04	0.06	0.01	0.07
V379A	-0.24	0.03	-0.21	-0.23	0.03	-0.19	-0.28	0.04	-0.25
V380A	-1.30	0.31	-0.99	-0.98	0.31	-0.67	-1.28	0.24	-1.04
H383A	-0.13	0.49	0.36	-0.40	0.49	0.09	-0.38	0.28	-0.10
E384A	-3.48	0.20	-3.28	-1.32	0.20	-1.12	-1.12	0.11	-1.01
H387A	-3.26	1.55	-1.72	-0.58	1.55	0.97	-0.95	1.76	0.81
F391A	-0.15	0.10	-0.05	-0.06	0.10	0.05	-0.25	0.10	-0.15
Y394A	-0.69	0.01	-0.68	-0.08	0.01	-0.07	-0.16	0.02	-0.14
R402A	0.10	0.00	0.10	0.11	0.00	0.11	0.12	0.00	0.12
E403A	-0.75	0.28	-0.47	-0.80	0.28	-0.53	-0.76	0.16	-0.60
N406A	-0.03	0.00	-0.02	-0.02	0.00	-0.02	-0.03	0.00	-0.02
P407A	0.01	0.01	0.02	-0.03	0.01	-0.02	-0.02	0.01	-0.01
H410A	-1.90	0.35	-1.56	-0.79	0.35	-0.45	-1.18	0.29	-0.89
E411A	-1.72	0.18	-1.54	-1.27	0.18	-1.09	-1.37	0.17	-1.20
D415A	-0.34	0.02	-0.32	-0.21	0.02	-0.20	-0.22	0.02	-0.20
D453A	0.04	0.01	0.05	0.04	0.01	0.05	0.06	0.00	0.07
K454A	-0.24	0.02	-0.22	-0.19	0.02	-0.17	-0.26	0.03	-0.23
F457A	-0.52	0.15	-0.37	-0.62	0.15	-0.47	-0.54	0.13	-0.41
F460A	-0.02	0.03	0.01	-0.05	0.03	-0.02	-0.03	0.03	0.00
K511A	-1.29	1.38	0.09	-1.02	1.38	0.36	-1.13	1.48	0.35
F512A	-1.62	0.29	-1.34	-1.44	0.29	-1.15	-1.74	0.24	-1.50
H513A	-3.11	0.80	-2.31	-2.98	0.80	-2.18	-3.02	0.86	-2.16
S516A	-0.08	0.02	-0.07	-0.13	0.02	-0.11	-0.09	0.03	-0.06
V518A	-1.65	0.42	-1.23	-1.77	0.42	-1.35	-1.77	0.22	-1.55
P519A	-0.11	0.10	-0.01	-0.27	0.10	-0.17	-0.19	0.02	-0.17

Y520A	-2.44	1.04	-1.40	-2.84	1.04	-1.80	-2.78	1.17	-1.61
R522A	-2.78	1.17	-1.61	-3.79	1.17	-2.63	-3.23	0.70	-2.53
Y523A	-4.26	0.99	-3.27	-5.44	0.99	-4.45	-5.13	0.99	-4.15
S526A	-0.04	0.02	-0.02	-0.03	0.02	-0.01	-0.07	0.01	-0.06
F527A	-1.18	0.37	-0.81	-1.31	0.37	-0.94	-1.04	0.41	-0.64
Q530A	-0.10	0.00	-0.09	-0.08	0.00	-0.08	-0.08	0.00	-0.07
Total	-43.63	13.50	-30.13	-38.87	13.50	-25.37	-39.54	12.19	-27.35

WFSW		Md1			Md2			Md3	
Mutation	ΔH	$-T\Delta S$	ΔG_{bind}	ΔH	$-T\Delta S$	ΔG_{bind}	ΔH	$-T\Delta S$	ΔG_{bind}
N66A	-0.10	0.04	-0.07	-0.27	0.13	-0.14	-0.07	0.05	-0.03
N70A	-0.43	0.06	-0.37	-0.39	0.13	-0.26	-0.41	0.12	-0.29
E143A	-0.51	0.06	-0.45	-0.50	0.06	-0.44	-0.46	0.05	-0.41
Q281A	-1.58	0.23	-1.35	-1.70	0.51	-1.19	-1.87	0.35	-1.52
T282A	-0.50	0.06	-0.44	-0.39	0.10	-0.29	-0.60	0.06	-0.54
V351A	-0.41	0.04	-0.37	-0.37	0.03	-0.33	-0.32	0.02	-0.30
H353A	-3.93	1.36	-2.57	-3.96	1.66	-2.30	-3.96	1.49	-2.46
S355A	-1.36	0.14	-1.22	-1.14	0.29	-0.85	-0.64	-0.35	-0.98
W357A	-3.42	3.91	0.49	-5.42	4.68	-0.73	-2.08	0.96	-1.11
D358A	-0.26	0.02	-0.24	-0.23	0.01	-0.22	-0.28	0.02	-0.26
Y360A	-0.09	0.04	-0.05	-0.59	0.15	-0.44	-0.13	0.05	-0.08
K368A	-0.06	0.02	-0.04	-0.05	0.01	-0.04	-0.01	0.01	0.00
E376A	-0.09	0.02	-0.07	0.06	0.03	0.09	0.04	0.01	0.05
V379A	-0.31	0.03	-0.28	-0.37	0.08	-0.29	-0.30	0.03	-0.26
V380A	-1.08	0.15	-0.93	-0.84	0.26	-0.58	-1.03	0.15	-0.89
H383A	-0.47	0.35	-0.12	-2.17	2.30	0.12	-0.46	0.27	-0.19
E384A	-1.37	0.18	-1.20	-1.56	1.08	-0.48	-1.37	0.19	-1.18
H387A	-0.86	2.27	1.41	-0.07	0.99	0.92	-0.72	1.52	0.80
F391A	-0.31	0.40	0.09	-0.31	0.20	-0.11	-0.24	0.26	0.02
Y394A	-0.19	0.05	-0.14	-0.06	0.02	-0.05	-0.15	0.03	-0.12
R402A	0.11	0.00	0.11	0.11	0.00	0.11	0.12	0.00	0.12
E403A	-0.95	0.18	-0.76	-1.04	0.79	-0.25	-0.83	0.24	-0.59
N406A	-0.02	0.00	-0.02	-0.02	0.00	-0.01	-0.02	0.00	-0.02
P407A	-0.02	0.02	-0.01	0.00	0.01	0.01	-0.02	0.01	-0.01
H410A	-1.54	0.63	-0.90	-0.42	0.07	-0.35	-1.20	0.37	-0.83
E411A	-1.23	0.24	-0.99	-1.37	0.30	-1.07	-1.22	0.17	-1.05
D415A	-0.25	0.02	-0.22	-1.05	0.24	-0.81	-0.24	0.02	-0.22
D453A	0.07	0.01	0.07	0.07	0.01	0.08	0.07	0.00	0.07
K454A	-0.30	0.03	-0.27	-0.35	0.04	-0.31	-0.29	0.02	-0.27
F457A	-0.54	0.13	-0.41	-0.51	0.21	-0.30	-0.49	0.12	-0.37
K511A	-1.29	1.31	0.03	-1.20	1.44	0.24	-1.07	1.39	0.32
F512A	-1.32	0.24	-1.08	-1.36	0.31	-1.05	-1.48	0.24	-1.24
H513A	-3.14	0.77	-2.37	-3.35	0.66	-2.70	-2.94	0.80	-2.14

S516A	-0.22	0.04	-0.18	-0.12	0.01	-0.11	-0.19	0.04	-0.16
V518A	-0.98	0.17	-0.82	-0.87	0.19	-0.68	-0.99	0.14	-0.85
Y520A	-2.52	1.20	-1.32	-2.61	1.13	-1.48	-2.57	1.20	-1.38
R522A	-2.84	0.47	-2.37	-0.75	0.04	-0.70	-3.07	0.51	-2.57
Y523A	-5.07	0.85	-4.22	-5.20	1.14	-4.06	-5.05	0.89	-4.16
S526A	-0.07	0.01	-0.05	-0.06	0.02	-0.04	-0.07	0.00	-0.07
F527A	-1.30	0.31	-0.99	-0.97	0.50	-0.46	-1.25	0.28	-0.97
Q530A	-0.08	0.00	-0.08	-0.18	0.01	-0.17	-0.09	0.00	-0.09
Total	-40.84	16.09	-24.75	-41.56	19.86	-21.70	-37.99	11.76	-26.22

WRFF		Md1			Md2			Md3	
Mutation	ΔH	$-T\Delta S$	ΔG_{bind}	ΔH	$-T\Delta S$	ΔG_{bind}	ΔH	$-T\Delta S$	ΔG_{bind}
W59A	0.18	0.05	0.23	0.32	0.15	0.47	0.18	0.14	0.32
Y62A	-1.52	0.65	-0.87	-1.55	0.36	-1.19	-1.28	0.36	-0.91
N66A	-1.60	0.72	-0.88	-1.50	0.38	-1.11	-1.10	0.36	-0.74
N70A	-1.03	1.11	0.08	-0.51	0.86	0.34	-0.75	0.92	0.17
E143A	-0.78	2.40	1.62	-0.81	2.37	1.56	-0.84	1.34	0.49
Q281A	-0.06	0.02	-0.04	-0.06	0.01	-0.05	-0.12	0.08	-0.04
T282A	-0.02	0.02	0.00	0.00	0.02	0.02	-0.02	0.00	-0.02
V351A	-0.33	0.12	-0.21	-0.35	0.00	-0.35	-0.43	0.05	-0.37
H353A	-3.20	0.65	-2.55	-3.99	0.74	-3.25	-1.17	0.30	-0.86
S355A	-0.65	1.04	0.39	-0.03	0.09	0.06	-1.38	0.95	-0.43
W357A	-2.16	0.89	-1.28	-1.29	0.44	-0.85	-2.40	0.90	-1.49
D358A	-0.19	0.14	-0.05	-0.02	0.05	0.03	-0.13	0.10	-0.04
F359A	0.00	0.00	0.00	0.07	0.01	0.08	-0.05	0.02	-0.02
Y360A	-1.11	0.53	-0.58	-0.70	2.06	1.36	-0.98	1.14	0.17
K368A	0.57	0.04	0.60	0.45	0.08	0.52	0.47	0.09	0.56
E376A	-0.39	0.06	-0.32	-0.28	0.02	-0.27	-0.69	0.51	-0.18
V379A	-0.04	0.00	-0.03	0.00	0.00	0.00	-0.13	0.07	-0.06
V380A	-0.67	0.14	-0.53	-0.02	0.00	-0.02	-0.57	0.39	-0.18
H383A	-1.19	1.07	-0.12	-0.28	0.28	0.00	-0.95	0.74	-0.21
E384A	-1.14	1.10	-0.04	-0.10	0.42	0.33	-3.27	1.41	-1.86
H387A	-0.47	0.48	0.02	-0.32	0.81	0.49	-0.67	0.97	0.30
F391A	-0.62	0.28	-0.34	-0.10	0.28	0.18	-0.40	0.39	-0.01
Y394A	-0.47	0.04	-0.43	-0.20	0.08	-0.12	-0.21	0.07	-0.14
E403A	-0.64	0.21	-0.43	-0.47	0.68	0.21	-0.39	0.35	-0.04
P407A	0.00	0.01	0.01	0.00	0.01	0.01	0.01	0.02	0.02
H410A	-1.29	0.22	-1.07	-0.72	0.31	-0.42	-1.37	0.42	-0.95
E411A	-0.85	0.09	-0.75	-0.80	0.11	-0.68	-1.43	0.22	-1.21
D415A	-0.07	0.02	-0.05	-0.01	0.02	0.01	-0.14	0.05	-0.09
D453A	-0.34	0.02	-0.33	-0.28	0.01	-0.27	-0.41	0.04	-0.37
K454A	0.29	0.01	0.30	0.26	0.01	0.27	0.26	0.01	0.27
K511A	0.37	0.03	0.40	0.24	0.01	0.25	0.46	0.12	0.58

F512A	-1.89	0.28	-1.61	-2.57	0.66	-1.91	-0.40	0.04	-0.35
H513A	-0.36	0.09	-0.28	-3.05	0.44	-2.61	-0.84	0.37	-0.47
S516A	-0.31	0.28	-0.03	-0.34	0.38	0.04	-0.04	0.15	0.11
V518A	-0.12	0.04	-0.08	-1.13	0.05	-1.08	-0.23	-0.03	-0.25
Y520A	-0.06	0.01	-0.05	-0.22	0.02	-0.20	-0.09	0.06	-0.03
R522A	0.07	0.09	0.16	-0.67	0.37	-0.30	-0.95	0.63	-0.32
Y523A	-0.40	0.16	-0.23	-1.36	0.25	-1.11	-0.99	0.22	-0.78
F527A	0.01	0.00	0.02	0.01	0.00	0.02	-0.01	0.01	0.00
Q530A	-0.01	0.01	0.00	0.00	0.01	0.00	-0.02	0.02	0.00
Total	-22.50	13.12	-9.37	-22.40	12.83	-9.57	-23.49	14.02	-9.46

WRQF		Md1			Md2			Md3	
Mutation	ΔH	$-T\Delta S$	ΔG_{bind}	ΔH	$-T\Delta S$	ΔG_{bind}	ΔH	$-T\Delta S$	ΔG_{bind}
Y62A	-0.06	0.05	-0.01	-1.62	0.70	-0.92	-2.73	1.11	-1.62
N66A	-0.07	0.06	-0.01	-1.01	0.13	-0.88	-0.66	0.27	-0.39
N70A	-0.08	0.02	-0.06	-2.31	1.38	-0.93	-0.04	0.02	-0.02
K118A	-0.30	0.16	-0.13	0.61	0.26	0.87	0.24	0.27	0.51
E143A	-0.25	0.02	-0.23	-0.50	0.07	-0.43	-0.22	0.03	-0.19
S222A	0.00	0.00	0.00	0.01	0.01	0.02	0.00	0.01	0.01
M223A	-0.72	0.27	-0.45	0.15	0.83	0.98	-0.06	0.34	0.28
V351A	-0.06	0.02	-0.04	-1.52	0.10	-1.42	-0.01	0.00	-0.01
H353A	-3.12	1.38	-1.74	-0.68	0.29	-0.39	-0.07	0.07	0.00
S355A	0.03	0.16	0.19	-2.07	1.46	-0.61	-0.53	0.49	-0.04
W357A	-1.44	0.72	-0.71	-9.50	3.46	-6.04	-5.13	5.03	-0.10
D358A	-0.53	0.04	-0.49	-0.62	0.28	-0.35	-0.44	0.03	-0.41
F359A	-0.02	0.01	-0.01	0.06	0.01	0.07	-0.06	0.00	-0.06
Y360A	-0.08	0.08	0.00	-3.19	0.81	-2.38	-1.47	0.27	-1.20
K368A	0.23	0.02	0.24	-0.96	1.18	0.22	0.14	0.01	0.15
V380A	0.01	0.00	0.02	-0.05	0.00	-0.05	-0.01	0.00	-0.01
H383A	-0.15	0.67	0.53	-0.22	0.28	0.06	-0.19	0.08	-0.10
E384A	-2.05	1.37	-0.68	-0.18	0.13	-0.06	-0.08	0.05	-0.02
H387A	-3.09	1.64	-1.45	-1.57	1.35	-0.22	-2.76	0.82	-1.95
F391A	-1.40	0.29	-1.11	-1.02	0.30	-0.72	-1.43	0.27	-1.16
E403A	-3.69	1.10	-2.58	-2.09	1.38	-0.72	-3.89	2.45	-1.44
N406A	-0.28	0.04	-0.25	-0.02	0.02	0.00	-0.22	0.03	-0.19
P407A	-1.42	0.33	-1.09	0.01	0.04	0.05	-1.69	0.16	-1.53
H410A	-3.92	1.00	-2.92	-0.84	0.47	-0.37	-2.35	0.29	-2.05
E411A	-1.39	0.13	-1.26	-0.77	0.14	-0.64	-1.08	0.11	-0.96
F512A	-0.78	0.23	-0.56	-1.32	0.22	-1.10	0.02	0.01	0.03
H513A	-2.22	0.55	-1.67	-0.67	0.10	-0.57	-0.03	0.01	-0.02
S516A	-0.06	0.06	0.00	-0.16	0.08	-0.07	-0.02	0.01	-0.01
V518A	-1.69	0.44	-1.25	-1.53	0.58	-0.95	-0.32	0.05	-0.27
R522A	-1.92	1.03	-0.89	-0.50	0.32	-0.18	-1.90	0.15	-1.74

Y523A	-1.32	0.33	-0.99	-0.46	0.14	-0.32	-0.10	0.03	-0.07
F570A	-0.83	0.14	-0.69	0.01	0.02	0.03	-0.56	0.20	-0.36
TOTAL	-32.66	12.36	-20.31	-34.57	16.52	-18.04	-27.63	12.68	-14.95

WRFV	Md1			Md2			Md3		
Mutation	ΔH	$-T\Delta S$	ΔG_{bind}	ΔH	$-T\Delta S$	ΔG_{bind}	ΔH	$-T\Delta S$	ΔG_{bind}
N66A	-0.07	0.08	0.01	-0.01	0.09	0.09	-0.04	0.10	0.06
N70A	-0.49	0.10	-0.39	-0.49	0.31	-0.18	-0.15	0.06	-0.10
E143A	-0.57	0.04	-0.53	-0.69	0.06	-0.63	-0.41	0.02	-0.40
L161A	-0.05	0.00	-0.04	-0.06	0.00	-0.06	-0.05	0.00	-0.05
E162A	-1.33	1.26	-0.08	-0.81	0.67	-0.14	-0.92	0.09	-0.83
T166A	-0.09	0.03	-0.06	-0.04	0.02	-0.02	-0.05	0.01	-0.04
W279A	-0.71	0.17	-0.53	-1.20	0.11	-1.09	-1.06	0.14	-0.93
Q281A	-3.35	0.68	-2.68	-3.41	0.49	-2.92	-3.45	0.55	-2.90
T282A	-0.07	0.01	-0.05	-0.07	0.03	-0.04	-0.09	0.03	-0.07
V351A	-0.25	0.02	-0.23	-0.50	0.04	-0.46	-0.06	0.00	-0.06
C313A	0.01	0.00	0.01	-0.03	0.01	-0.01	0.00	0.00	0.00
H353A	-4.61	1.52	-3.09	-2.08	1.65	-0.43	-4.18	2.10	-2.09
S355A	-0.79	-0.50	-1.29	-0.42	0.02	-0.40	-0.45	-0.28	-0.74
W357A	-1.52	1.32	-0.20	-0.55	0.34	-0.21	-1.29	0.77	-0.52
D358A	-0.43	0.02	-0.41	-0.39	0.02	-0.37	-0.48	0.02	-0.46
K368A	0.36	0.01	0.37	0.20	0.03	0.23	0.36	0.01	0.37
Q369A	-0.70	0.25	-0.45	-0.22	0.21	-0.01	-0.81	0.71	-0.10
C370A	-0.02	0.03	0.01	-0.01	0.05	0.04	0.00	0.02	0.02
V379A	-0.01	0.00	-0.01	-0.05	0.00	-0.05	-0.08	0.00	-0.07
V380A	-0.27	0.47	0.20	-1.05	0.22	-0.83	-1.07	0.52	-0.56
H383A	-0.37	0.39	0.02	-0.26	0.22	-0.04	-0.29	0.19	-0.10
E384A	-1.38	0.23	-1.15	-0.61	0.13	-0.48	-2.34	0.43	-1.91
H387A	-1.38	1.11	-0.27	-1.29	0.83	-0.46	-1.04	1.30	0.27
F391A	-0.46	0.40	-0.06	-0.08	0.03	-0.06	-1.00	0.73	-0.26
Y394A	-0.14	0.01	-0.12	-0.03	0.01	-0.02	-0.47	0.04	-0.43
E403A	-1.42	0.25	-1.17	-0.83	0.21	-0.62	-0.98	0.21	-0.77
N406A	-0.05	0.01	-0.04	-0.03	0.00	-0.03	-0.03	0.01	-0.02
P407A	-0.33	0.10	-0.22	-0.04	0.00	-0.03	0.00	0.01	0.02
H410A	-2.23	0.94	-1.30	-0.66	0.12	-0.55	-1.28	0.34	-0.94
E411A	-2.19	0.33	-1.86	-1.74	0.12	-1.62	-1.55	0.15	-1.40
D415A	-0.08	0.01	-0.07	-0.06	0.02	-0.04	-0.07	0.03	-0.03
K454A	0.22	0.01	0.23	0.25	0.02	0.27	0.24	0.01	0.25
F457A	-1.16	0.22	-0.93	-1.32	0.12	-1.20	-1.26	0.11	-1.15
F460A	-0.14	0.02	-0.12	-0.08	0.02	-0.06	-0.10	0.02	-0.07
K511A	-0.57	1.64	1.07	-0.58	1.71	1.13	-0.47	1.83	1.36
F512A	-1.65	0.23	-1.42	-0.99	0.40	-0.59	-1.25	0.45	-0.80
H513A	-3.56	0.83	-2.73	-4.20	0.86	-3.34	-3.92	0.76	-3.16

S516A	-0.14	0.03	-0.11	-0.10	0.11	0.01	-0.07	0.02	-0.05
V518A	-0.73	0.07	-0.66	-0.89	0.27	-0.62	-1.13	0.17	-0.96
Y520A	-3.45	1.05	-2.41	-3.44	0.74	-2.70	-3.43	0.80	-2.63
R522A	-0.98	0.13	-0.85	-4.09	0.50	-3.59	-0.99	0.20	-0.79
Y523A	-2.94	1.55	-1.39	-3.87	1.36	-2.51	-2.59	0.95	-1.63
S526A	-0.03	0.02	-0.01	-0.03	0.01	-0.03	-0.02	0.01	-0.01
F527A	-0.39	0.05	-0.34	-0.51	0.03	-0.48	-0.47	0.03	-0.44
Total	-40.50	15.14	-25.36	- 37.35	12.19	-25.16	- 38.78	13.68	-25.11

RWWD	Md1			Md2			Md3		
Mutation	ΔH	$-T\Delta S$	ΔG_{bind}	ΔH	$-T\Delta S$	ΔG_{bind}	ΔH	$-T\Delta S$	ΔG_{bind}
Y62A	-0.09	0.26	0.17	-0.65	0.20	-0.45	-0.63	1.22	0.60
N66A	-0.15	0.13	-0.02	-0.84	0.28	-0.56	-1.02	1.74	0.72
Y69A	-0.03	0.01	-0.02	-0.06	0.00	-0.05	0.01	0.25	0.25
N70A	-0.13	0.07	-0.05	-0.33	0.09	-0.24	-0.49	0.60	0.12
E143A	-0.20	0.06	-0.14	-0.20	0.08	-0.13	0.15	0.34	0.49
W279A	-0.12	0.05	-0.07	0.05	0.02	0.08	-0.83	0.16	-0.67
Q281A	-0.31	0.53	0.22	0.08	0.12	0.20	-0.61	0.48	-0.13
V351A	-0.06	0.00	-0.06	-0.11	0.01	-0.10	0.05	0.05	0.10
H353A	-2.53	0.35	-2.18	-2.12	1.25	-0.88	-5.00	1.45	-3.55
S355A	-0.44	-0.20	-0.64	-0.95	0.82	-0.13	-0.43	0.65	0.22
W357A	-0.50	0.40	-0.10	-3.56	1.08	-2.48	-0.59	0.70	0.11
D358A	0.03	-0.02	0.01	0.20	0.02	0.22	0.11	0.03	0.14
F359A	-0.02	0.00	-0.01	0.02	0.00	0.02	0.11	0.01	0.13
Y360A	-0.85	0.42	-0.43	-0.13	0.06	-0.06	-0.52	0.23	-0.29
K368A	0.23	0.03	0.26	0.15	0.03	0.18	-0.24	0.12	-0.12
V380A	-0.30	0.48	0.17	0.11	0.34	0.45	-0.18	0.11	-0.07
H383A	-0.27	0.95	0.68	-3.64	1.80	-1.84	-1.39	2.48	1.09
E384A	-3.42	0.74	-2.68	-1.35	1.49	0.14	-0.44	1.21	0.76
H387A	-0.53	0.88	0.35	0.33	0.82	1.15	0.29	0.26	0.55
F391A	-0.54	0.30	-0.24	0.07	0.05	0.12	-0.22	0.11	-0.11
Y394A	-0.36	0.04	-0.33	-0.01	0.09	0.08	-0.04	0.00	-0.03
R402A	-0.25	0.01	-0.24	-0.22	0.01	-0.21	-0.07	0.01	-0.06
E403A	0.28	0.52	0.81	0.31	0.20	0.50	0.05	0.04	0.09
N406A	-0.01	0.03	0.03	0.00	0.04	0.04	-0.02	0.01	-0.01
P407A	0.06	0.21	0.27	0.14	0.25	0.39	0.01	0.03	0.04
H410A	-1.26	0.31	-0.95	-1.29	0.98	-0.31	-0.14	0.13	-0.02
E411A	-0.44	0.23	-0.21	-0.93	0.29	-0.64	-1.81	0.89	-0.91
F457A	-1.16	0.69	-0.47	0.01	0.02	0.04	-0.72	0.32	-0.41
F460A	-0.22	0.04	-0.18	0.02	0.01	0.03	-0.03	0.01	-0.03
K511A	0.64	0.15	0.79	0.19	0.14	0.33	-1.11	0.22	-0.89
F512A	-0.11	0.04	-0.08	-1.48	0.34	-1.14	-0.15	0.24	0.08

H513A	-2.26	0.56	-1.70	-1.07	0.23	-0.83	-1.48	0.48	-1.00
S516A	-0.01	0.05	0.04	-0.09	0.06	-0.03	-0.02	0.05	0.03
V518A	-0.86	0.83	-0.03	-0.95	0.40	-0.56	-2.09	0.19	-1.89
Y520A	-1.49	1.04	-0.45	-0.05	0.01	-0.04	-0.71	0.23	-0.48
R522A	-2.88	2.35	-0.53	-2.59	1.79	-0.80	-1.64	0.32	-1.32
Y523A	-2.19	1.23	-0.96	-0.94	0.48	-0.46	-3.29	1.05	-2.24
Total	-22.74	13.77	-8.97	-21.88	13.91	-7.97	-25.11	16.40	-8.71

YYWK		Md1			Md2			Md3	
Mutation	ΔH	$-T\Delta S$	ΔG_{bind}	ΔH	$-T\Delta S$	ΔG_{bind}	ΔH	$-T\Delta S$	ΔG_{bind}
W59A	0.11	0.17	0.28	0.03	0.11	0.14	0.05	0.06	0.11
Y62A	-0.54	0.74	0.20	-0.37	0.30	-0.07	-0.19	0.07	-0.12
E64A	-0.24	0.02	-0.22	-0.19	0.02	-0.18	-0.23	0.01	-0.22
N66A	-1.04	0.71	-0.34	-1.12	0.56	-0.56	-0.47	0.72	0.25
W67A	-0.09	0.00	-0.09	-0.03	0.00	-0.02	-0.19	0.01	-0.18
N70A	-0.38	0.07	-0.31	-0.34	0.20	-0.14	-1.19	0.73	-0.46
E143A	-0.34	0.04	-0.30	-0.40	0.05	-0.35	-0.55	0.17	-0.38
W279A	-0.08	0.07	-0.01	-0.15	0.02	-0.13	-0.09	0.01	-0.08
Q281A	-0.06	0.02	-0.03	-0.15	0.03	-0.12	-0.06	0.01	-0.05
V351A	-0.22	0.01	-0.22	-0.31	0.01	-0.30	-0.22	0.03	-0.19
H353A	-2.83	0.43	-2.40	-3.18	1.57	-1.61	-3.34	1.05	-2.29
S355A	-0.47	-0.28	-0.75	-0.50	-0.33	-0.83	-0.46	-0.25	-0.70
W357A	-1.84	0.23	-1.61	-2.04	0.20	-1.84	-5.88	1.30	-4.59
D358A	-1.02	1.12	0.09	-0.93	0.35	-0.58	-0.68	0.13	-0.55
Y360A	-2.56	0.84	-1.72	-2.63	1.84	-0.79	-2.90	1.12	-1.78
K368A	0.33	0.02	0.35	0.29	0.03	0.32	0.37	0.02	0.39
V380A	-0.20	0.19	-0.02	-0.04	0.03	-0.01	-0.05	0.00	-0.04
H383A	-0.26	0.38	0.12	-0.22	0.48	0.26	0.17	0.54	0.71
E384A	-1.00	0.32	-0.68	-0.51	0.30	-0.21	-0.45	0.15	-0.30
H387A	-0.74	0.77	0.03	-0.94	0.92	-0.02	-0.59	1.21	0.62
F391A	-3.78	1.30	-2.49	-1.55	0.39	-1.15	-1.90	0.97	-0.93
Y394A	-1.67	0.57	-1.10	-0.44	0.19	-0.26	-1.35	0.18	-1.16
R402A	0.28	0.00	0.28	0.31	0.05	0.36	0.21	0.03	0.24
E403A	-1.44	1.46	0.02	-1.85	0.57	-1.28	-0.63	0.48	-0.15
P407A	-0.20	0.04	-0.16	-0.16	0.03	-0.13	0.02	0.01	0.03
H410A	-2.29	0.25	-2.04	-2.38	0.39	-2.00	-0.83	0.26	-0.57
E411A	-1.59	0.79	-0.79	-1.72	0.71	-1.00	-1.47	0.59	-0.88
F457A	-0.01	0.01	-0.01	-0.02	0.01	-0.02	-0.01	0.00	0.00
K511A	0.23	0.04	0.27	-0.12	0.14	0.01	0.01	0.04	0.05
F512A	-1.76	0.33	-1.42	-1.64	0.98	-0.66	-1.37	0.98	-0.39
H513A	-1.06	0.15	-0.91	-2.22	0.50	-1.72	-2.45	0.68	-1.77
S516A	-0.06	0.01	-0.05	-0.04	0.02	-0.02	-0.22	0.30	0.09
V518A	-0.37	0.07	-0.30	-0.26	0.01	-0.25	-0.75	0.17	-0.58

Y520A	-0.10	0.03	-0.07	-0.20	0.03	-0.17	-0.17	0.01	-0.16
R522A	-1.47	0.42	-1.05	-1.42	0.28	-1.14	-1.29	0.45	-0.84
Y523A	-0.96	0.11	-0.85	-1.50	0.23	-1.27	-0.95	0.22	-0.73
Total	-29.72	11.45	-18.27	-28.94	11.22	-17.72	-30.09	12.47	-17.62

WWDW		Md1			Md2			Md3	
Mutation	ΔH	$-T\Delta S$	ΔG_{bind}	ΔH	$-T\Delta S$	ΔG_{bind}	ΔH	$-T\Delta S$	ΔG_{bind}
N66A	-0.11	0.01	-0.09	-0.12	0.01	-0.11	-0.15	0.01	-0.14
N70A	-0.12	0.02	-0.10	-0.12	0.02	-0.10	-0.22	0.02	-0.21
E143A	-0.11	0.02	-0.09	-0.05	0.02	-0.03	-0.02	0.02	-0.01
Q281A	-1.70	0.49	-1.21	-1.92	0.71	-1.21	-1.68	0.67	-1.01
T282A	-0.51	0.16	-0.35	-0.50	0.18	-0.33	-0.48	0.16	-0.32
V351A	-0.10	0.00	-0.10	-0.10	0.00	-0.10	-0.11	0.01	-0.10
H353A	-4.67	2.30	-2.37	-4.29	1.42	-2.87	-4.39	1.68	-2.70
S355A	-0.33	-0.04	-0.37	-0.18	0.01	-0.17	-0.48	0.35	-0.13
W357A	-2.27	0.32	-1.94	-2.91	0.27	-2.64	-3.11	0.36	-2.75
D358A	-0.03	0.00	-0.02	-0.05	0.01	-0.05	-0.06	0.01	-0.05
Y360A	-0.06	0.01	-0.04	-0.26	0.02	-0.23	-0.33	0.03	-0.30
K368A	-0.44	0.03	-0.41	-0.43	0.02	-0.41	-0.39	0.02	-0.38
E376A	0.42	0.08	0.50	0.40	0.02	0.43	0.41	0.09	0.50
V379A	-0.26	0.13	-0.13	-0.15	0.04	-0.11	-0.07	0.04	-0.03
V380A	-0.28	0.24	-0.04	-0.31	0.31	0.00	-0.56	0.22	-0.34
H383A	1.17	0.72	1.89	0.53	0.26	0.79	0.05	1.18	1.23
E384A	-0.66	1.66	1.00	-3.37	0.76	-2.61	-3.66	1.14	-2.52
H387A	-1.00	2.24	1.24	-3.41	1.19	-2.22	-3.11	2.56	-0.55
F391A	0.16	0.16	0.32	0.03	0.30	0.33	-0.18	0.35	0.18
Y394A	-0.10	0.01	-0.10	-0.13	0.01	-0.12	-0.16	0.01	-0.15
R402A	-0.06	0.00	-0.06	-0.05	0.00	-0.05	-0.05	0.00	-0.05
E403A	-1.16	0.22	-0.93	-0.84	0.52	-0.32	-0.70	0.55	-0.15
N406A	-0.05	0.00	-0.05	-0.02	0.00	-0.01	-0.01	0.00	-0.01
P407A	-0.43	0.10	-0.33	-0.03	0.01	-0.01	0.00	0.01	0.01
H410A	-1.70	0.30	-1.40	-1.60	0.32	-1.27	-1.54	0.34	-1.20
E411A	-1.20	0.31	-0.89	-0.91	0.16	-0.76	-0.49	0.13	-0.36
D415A	-1.07	0.19	-0.87	-0.16	0.09	-0.07	-0.43	0.07	-0.36
D453A	0.36	0.00	0.37	0.39	0.02	0.40	0.37	0.00	0.38
K454A	-0.75	0.06	-0.69	-0.61	0.09	-0.53	-0.64	0.05	-0.59
F457A	-0.07	0.26	0.19	-0.16	0.27	0.11	-0.12	0.27	0.15
K511A	-2.10	1.09	-1.01	-1.33	1.66	0.33	-1.63	1.41	-0.22
F512A	-0.94	0.17	-0.77	-1.18	0.24	-0.94	-1.18	0.26	-0.92
H513A	-3.47	0.59	-2.87	-3.04	0.85	-2.19	-3.19	0.66	-2.53
S516A	-0.18	0.03	-0.15	-0.19	0.02	-0.17	-0.08	0.01	-0.06
V518A	-1.94	0.31	-1.63	-1.80	0.26	-1.55	-1.36	0.43	-0.93
P519A	-0.18	0.01	-0.17	-0.13	0.01	-0.11	-0.11	0.01	-0.10

Y520A	-2.88	0.94	-1.94	-2.89	0.99	-1.90	-2.62	1.14	-1.48
R522A	-3.52	0.48	-3.04	-2.56	0.15	-2.40	-2.54	0.18	-2.36
Y523A	-4.98	1.53	-3.45	-4.96	2.02	-2.93	-4.38	1.59	-2.78
S526A	-0.07	0.03	-0.04	-0.01	0.01	0.00	-0.08	0.09	0.02
F527A	-0.72	0.41	-0.31	-0.67	0.44	-0.22	-0.81	0.40	-0.41
Q530A	-0.16	0.01	-0.16	-0.08	0.01	-0.07	-0.09	0.01	-0.08
Total	-38.24	15.61	-22.63	-40.16	13.74	-26.42	-40.35	16.54	-23.81

WWTY		Md1			Md2			Md3	
Mutation	ΔH	$-T\Delta S$	ΔG_{bind}	ΔH	$-T\Delta S$	ΔG_{bind}	ΔH	$-T\Delta S$	ΔG_{bind}
N66A	-0.14	0.03	-0.11	-0.09	0.09	0.00	-0.12	0.02	-0.10
N70A	-0.14	0.05	-0.09	-0.25	0.10	-0.14	-0.15	0.03	-0.13
E143A	-0.36	0.03	-0.34	-0.42	0.04	-0.38	-0.42	0.03	-0.40
Q281A	-3.65	0.61	-3.04	-1.98	0.51	-1.24	-2.18	0.92	-1.26
T282A	-0.12	0.00	-0.12	-0.53	0.07	-0.45	-0.39	0.09	-0.30
V351A	-0.09	0.00	-0.09	-0.11	0.00	-0.11	-0.11	0.00	-0.10
H353A	-3.59	1.81	-1.78	-3.93	1.29	-2.81	-3.82	1.47	-2.34
S355A	-0.74	-0.49	-1.23	-0.70	-0.41	-0.99	-0.21	-0.07	-0.28
W357A	-5.04	2.47	-2.57	-2.14	1.17	-0.09	-3.08	0.83	-2.25
D358A	-0.33	0.04	-0.29	-0.32	0.05	-0.26	-0.25	0.01	-0.24
Y360A	-0.13	0.04	-0.09	-0.09	0.04	-0.05	-0.33	0.04	-0.29
K368A	0.00	0.02	0.02	-0.03	0.03	0.00	-0.02	0.01	-0.01
V379A	-0.15	0.07	-0.08	-0.16	0.03	-0.12	-0.28	0.08	-0.21
V380A	-1.21	0.17	-1.04	-0.97	0.30	-0.71	-1.15	0.18	-0.97
H383A	-0.11	0.24	0.13	-0.11	0.30	0.29	-0.79	1.31	0.52
E384A	-1.50	0.25	-1.24	-2.27	0.28	-1.95	-2.42	0.84	-1.59
H387A	-1.44	0.47	-0.97	-1.21	1.20	0.33	-2.96	3.15	0.19
F391A	-1.19	1.11	-0.08	-1.32	1.32	-0.07	-0.40	0.27	-0.13
Y394A	-0.38	0.12	-0.25	-0.51	0.08	-0.43	-0.10	0.01	-0.09
R402A	0.10	0.00	0.10	0.09	0.01	0.10	0.11	0.00	0.11
E403A	-0.98	0.15	-0.83	-0.66	0.10	-0.56	-1.17	0.52	-0.65
N406A	-0.03	0.00	-0.03	-0.02	0.00	-0.02	-0.02	0.00	-0.02
P407A	-0.03	0.01	-0.03	0.00	0.01	0.00	-0.02	0.02	-0.01
H410A	-1.98	0.29	-1.69	-1.66	0.33	-1.33	-1.58	0.69	-0.90
E411A	-1.23	0.09	-1.14	-0.89	0.10	-0.77	-1.50	0.21	-1.29
D415A	-0.42	0.03	-0.39	-0.23	0.02	-0.21	-0.73	0.09	-0.63
D453A	-0.27	0.02	-0.25	-0.26	0.04	-0.22	-0.32	0.05	-0.27
F457A	-0.85	0.10	-0.75	-0.57	0.18	-0.39	-0.47	0.16	-0.31
K511A	-1.30	1.64	0.34	-0.83	1.69	0.55	-0.79	1.67	0.89
F512A	-1.12	0.29	-0.83	-1.49	0.24	-1.28	-1.40	0.24	-1.16
H513A	-3.21	0.93	-2.28	-3.15	0.75	-2.50	-3.31	0.69	-2.62
S516A	-0.21	0.03	-0.18	-0.19	0.03	-0.16	-0.20	0.02	-0.18
V518A	-2.28	0.21	-2.07	-1.63	0.25	-1.39	-1.68	0.31	-1.37

P519A	-0.13	0.01	-0.12	-0.10	0.01	-0.09	-0.12	0.01	-0.11
Y520A	-3.05	1.02	-2.02	-2.36	1.23	-1.41	-2.51	1.13	-1.38
R522A	-1.92	0.15	-1.77	-1.80	0.44	-1.13	-1.85	0.20	-1.65
Y523A	-5.55	0.80	-4.75	-5.40	0.79	-4.74	-5.08	1.02	-4.06
S526A	-0.03	0.00	-0.02	-0.04	0.01	-0.03	-0.06	0.02	-0.04
F527A	-0.52	0.29	-0.23	-1.11	0.28	-0.84	-0.94	0.23	-0.72
Q530A	-0.08	0.00	-0.08	-0.08	0.01	-0.06	-0.13	0.01	-0.12
Total	-45.38	13.11	-32.27	-39.51	13.02	-25.67	-42.98	16.53	-26.46

RRYQ		Md1			Md2			Md3	
Mutation	ΔH	$-T\Delta S$	ΔG_{bind}	ΔH	$-T\Delta S$	ΔG_{bind}	ΔH	$-T\Delta S$	ΔG_{bind}
Y62A	-0.91	0.48	-0.44	-0.06	0.01	-0.05	-0.33	0.15	-0.18
N66A	-0.64	0.66	0.03	-0.08	0.05	-0.03	-0.62	0.50	-0.12
Y69A	-0.05	0.12	0.07	-0.05	0.03	-0.02	-0.06	0.01	-0.04
N70A	-0.17	0.10	-0.06	-0.09	0.07	-0.02	-0.30	0.26	-0.04
E143A	-1.04	0.08	-0.96	-1.08	2.01	0.93	-0.97	0.44	-0.52
W279A	0.01	0.05	0.06	0.01	0.02	0.03	0.00	0.04	0.04
Q281A	0.03	0.09	0.12	0.09	0.35	0.44	0.04	0.07	0.11
V351A	-0.06	0.01	-0.05	-0.22	0.00	-0.21	-0.09	0.02	-0.07
H353A	-1.93	0.35	-1.58	-3.93	2.87	-1.06	-2.54	2.21	-0.33
S355A	-0.22	-0.25	-0.47	-0.04	0.08	0.03	-0.85	0.80	-0.05
W357A	-4.23	0.93	-3.29	-0.95	0.84	-0.12	-3.02	0.81	-2.21
D358A	-0.63	-0.01	-0.64	-0.39	0.03	-0.36	-0.34	0.00	-0.34
F359A	-0.40	0.12	-0.28	0.01	0.00	0.01	-0.09	0.01	-0.08
Y360A	-1.76	0.47	-1.29	-0.02	0.01	-0.01	-0.14	0.10	-0.04
K368A	0.84	0.03	0.88	0.53	0.07	0.61	0.64	0.10	0.74
V380A	0.13	0.43	0.56	-0.39	0.17	-0.22	-0.45	0.30	-0.15
H383A	-3.55	1.40	-2.15	-3.73	1.16	-2.58	-4.83	2.00	-2.83
E384A	-3.01	1.23	-1.78	-0.45	0.25	-0.20	-1.64	1.00	-0.64
H387A	-1.82	0.90	-0.92	-0.81	0.78	-0.03	-1.21	0.84	-0.37
F391A	-0.76	0.85	0.09	0.05	0.02	0.07	-0.35	0.17	-0.18
Y394A	-0.23	0.17	-0.06	-0.02	0.00	-0.02	-0.07	0.01	-0.06
E403A	-0.68	1.00	0.32	-0.58	0.12	-0.45	-0.53	0.35	-0.18
P407A	-0.06	0.02	-0.03	0.00	0.01	0.01	-0.09	0.13	0.04
H410A	-1.57	0.31	-1.25	-0.31	0.06	-0.25	-1.99	0.53	-1.46
E411A	-2.01	0.18	-1.83	-2.34	0.15	-2.19	-3.84	0.59	-3.25
F457A	-0.06	0.05	-0.01	-0.17	0.30	0.13	-0.05	0.04	-0.01
F460A	0.01	0.00	0.01	0.01	0.02	0.03	0.00	0.01	0.01
K511A	0.90	0.09	0.99	0.70	0.21	0.91	0.89	0.30	1.18
F512A	-0.10	0.27	0.17	-0.22	0.01	-0.20	-0.12	0.07	-0.05
H513A	-0.02	0.26	0.25	-1.66	0.78	-0.88	-0.39	0.36	-0.03
S516A	-0.03	0.18	0.15	-0.01	0.01	0.00	-0.02	0.00	-0.01
V518A	0.06	0.03	0.09	-1.33	0.51	-0.83	-0.87	0.21	-0.66

Y520A	-0.05	0.06	0.02	-0.17	0.06	-0.11	-0.03	0.08	0.05
R522A	0.23	0.15	0.38	-3.85	0.53	-3.32	-0.72	0.48	-0.24
Y523A	-1.05	0.34	-0.71	-4.03	0.94	-3.09	-1.52	0.34	-1.18
F527A	-0.09	0.03	-0.06	-0.32	0.21	-0.11	-0.20	0.04	-0.15
TOTAL	-24.91	11.20	-13.71	-25.89	12.74	-13.15	-26.68	13.36	-13.33

GGSG		Md1			Md2			Md3	
Mutation	ΔH	$-T\Delta S$	ΔG_{bind}	ΔH	$-T\Delta S$	ΔG_{bind}	ΔH	$-T\Delta S$	ΔG_{bind}
E162A	0.00	0.06	0.06	0.16	0.06	0.22	-0.01	0.08	0.07
W279A	-0.56	0.06	-0.50	-0.25	0.08	-0.16	-0.60	0.08	-0.52
Q281A	-2.00	1.18	-0.82	-0.64	0.29	-0.36	-2.41	0.84	-1.57
H353A	-1.86	1.91	0.05	-2.65	2.30	-0.35	-2.93	3.77	0.84
S355A	-0.64	1.19	0.55	-0.24	0.19	-0.06	-0.48	1.62	1.14
Q369A	-0.07	0.01	-0.06	-0.08	0.05	-0.02	-0.07	0.01	-0.06
V379A	-0.01	0.00	0.00	0.00	0.00	0.00	-0.07	0.01	-0.06
V380A	-0.24	0.10	-0.14	-0.25	0.08	-0.17	-0.64	0.15	-0.49
H383A	-1.08	1.17	0.10	0.20	1.18	1.38	-0.85	1.96	1.11
E384A	-1.83	1.06	-0.77	-2.16	1.39	-0.77	-1.67	0.66	-1.02
H387A	0.24	1.65	1.89	-0.88	4.23	3.35	0.64	0.73	1.38
F391A	0.12	0.03	0.15	0.04	0.18	0.22	0.10	0.01	0.11
E411A	-0.81	0.30	-0.51	-0.57	0.25	-0.32	-0.59	0.39	-0.20
K511A	-1.26	1.45	0.19	-1.10	1.16	0.06	-1.06	1.55	0.49
F512A	0.01	0.02	0.03	-0.06	0.03	-0.03	-0.10	0.17	0.06
H513A	-0.86	1.08	0.23	-2.04	1.12	-0.91	-1.67	1.56	-0.12
V518A	0.13	0.01	0.13	0.09	0.00	0.09	0.10	-0.01	0.09
Y520A	-1.93	1.14	-0.79	-1.95	1.24	-0.71	-1.77	1.22	-0.55
R522A	-0.22	0.14	-0.08	-0.13	0.12	-0.02	-0.12	0.14	0.02
Y523A	-4.22	1.23	-3.00	-3.59	0.96	-2.64	-3.83	1.01	-2.82
TOTAL	-17.08	13.79	-3.29	-16.11	14.90	-1.21	-18.05	15.94	-2.11

SGGG		Md1			Md2			Md3	
Mutation	ΔH	$-T\Delta S$	ΔG_{bind}	ΔH	$-T\Delta S$	ΔG_{bind}	ΔH	$-T\Delta S$	ΔG_{bind}
Q281A	-0.11	0.15	0.04	-0.05	0.06	0.01	-0.17	0.09	-0.07
T282A	-0.40	0.23	-0.17	-0.01	0.02	0.01	-0.32	0.08	-0.24
H353A	-1.02	0.59	-0.43	-1.20	0.71	-0.49	-2.08	1.33	-0.75
S355A	-0.03	0.04	0.01	-0.04	0.07	0.03	-0.04	0.03	-0.01
V379A	0.12	0.14	0.27	0.31	-0.03	0.28	1.12	0.17	1.29
V380A	-0.97	0.23	-0.75	-0.63	0.41	-0.22	-1.11	0.21	-0.90
H383A	-0.28	3.60	3.32	-0.52	3.46	2.94	-0.20	1.54	1.34
E384A	-1.18	2.11	0.93	-1.13	1.94	0.81	-1.00	0.82	-0.18
H387A	3.31	1.48	4.79	3.07	1.52	4.59	3.07	1.09	4.16
E411A	0.64	0.12	0.76	0.33	0.45	0.78	0.60	0.08	0.68

D415A	-1.03	0.23	-0.80	-0.54	1.84	1.29	-0.94	0.22	-0.72
D453A	-0.07	0.16	0.09	0.07	0.16	0.23	0.34	0.09	0.43
F457A	-0.25	0.08	-0.17	-0.09	0.01	-0.09	-0.75	0.06	-0.69
F512A	0.09	0.02	0.10	-0.10	0.13	0.03	0.03	0.02	0.05
H513A	-0.22	0.27	0.06	-1.00	0.62	-0.38	-0.50	0.19	-0.31
V518A	0.07	0.01	0.08	0.02	0.02	0.04	-0.10	0.02	-0.08
R522A	-0.59	0.27	-0.32	-0.34	0.12	-0.22	-1.03	1.18	0.15
Y523A	-0.87	2.05	1.18	-1.21	0.92	-0.29	-1.97	1.65	-0.32
S526A	-0.05	0.02	-0.03	-0.03	0.04	0.01	-0.07	0.03	-0.04
F527A	-0.59	0.20	-0.39	-0.20	0.25	0.05	-0.92	0.12	-0.81
Q530A	-0.07	0.03	-0.04	0.02	0.06	0.07	-0.09	0.03	-0.06
TOTAL	-3.49	12.05	8.55	-3.26	12.75	9.49	-6.12	9.04	2.92
